# Left Main Coronary Artery Disease and Outcomes after Percutaneous Coronary Intervention for Chronic Total Occlusions

**DOI:** 10.3390/jcm9040938

**Published:** 2020-03-30

**Authors:** Max-Paul Winter, Georg Goliasch, Philipp Bartko, Jolanta Siller-Matula, Mohamed Ayoub, Stefan Aschauer, Klaus Distelmaier, Catherine Gebhard, Kambis Mashayekhi, Miroslaw Ferenc, Christian Hengstenberg, Aurel Toma

**Affiliations:** 1 Department of Internal Medicine II, Division of Cardiology, Medical University of Vienna, 1090 Vienna, Austria; max-paul.winter@meduniwien.ac.at (M.-P.W.); georg.goliasch@meduniwien.ac.at (G.G.); philippemanuel.bartko@meduniwien.ac.at (P.B.); jolanta.siller-matula@meduniwien.ac.at (J.S.-M.); stefan.aschauer@meduniwien.ac.at (S.A.); klaus.distelmaier@meduniwien.ac.at (K.D.); catherine.gebhard@meduniwien.ac.at (C.G.); christian.hengstenberg@meduniwien.ac.at (C.H.); 2 Division of Cardiology and Angiology II, University Heart Center Freiburg-Bad Krozingen, 79189 Bad Krozingen, Germany; mohamed.ayoub@universitaets-herzzentrum.de (M.A.); kambis.mashayekhi@universitaets-herzzentrum.de (K.M.); miroslaw.ferenc@universitaets-herzzentrum.de (M.F.)

**Keywords:** chronic total occlusion, CTO, PCI, coronary artery disease

## Abstract

Background: Concomitant left main coronary artery (LMCA) disease in patients with chronic total occlusions (CTO) commonly results in referral for coronary artery bypass grafting, although the impact of LMCA in CTO patients remains largely unknown. Nevertheless, patient selection for percutaneous coronary intervention of CTOs (CTO-PCI) or alternative revascularization strategies should be based on precise evaluation of the coronary anatomy to anticipate those patients that most likely benefit from a procedure and not on strict adherence to perpetual clinical practice. Therefore, the aim of this study was to assess the impact of LMCA disease on long-term outcomes in patients undergoing percutaneous coronary intervention for CTO. Methods: We enrolled 3860 consecutive patients undergoing PCI for at least one CTO lesion and investigated the predictive value of concomitant LMCA disease. All-cause mortality was defined as the primary study endpoint. Results: We observed that LMCA disease is significantly associated with mortality. In the Cox regression analysis, we observed a crude hazard ratio (HR) 1.59 (95% confidence interval (CI) 1.23–2.04, *p* < 0.001) for patients with LMCA disease as compared to patients without. Results remained unchanged after bootstrap- or clinical confounder-based adjustment. Conclusion: LMCA disease is associated with excess mortality in CTO patients. Specifically, anatomical features such as CTO of the circumflex artery represent a high risk patient population.

## 1. Introduction

Coronary artery chronic total occlusions (CTO) represent the most advanced form of coronary artery disease (CAD), which are defined by coronary arteries with absent anterograde blood flow for more than 3 months [1]. As compared to non-CTO percutaneous coronary intervention (PCI), CTO-PCI is more complex, including risky crossing techniques, multiple stenting, and partly overlapping stents. For proper selection of patients that are suitable for these procedures, the meticulous evaluation of the coronary anatomy is paramount, and in those cases, CTO revascularization harbors significant clinical and even survival benefits [2,3,4].

Previous observations suggest that the prognostic benefits of CTO recanalization not only depend on the extent of coronary artery disease, but there is a variable benefit depending upon the target vessel. There is certain evidence that CTO of the left anterior descending (LAD) and the circumflex (CX) artery harbors a survival benefit, underpinning the prognostic significance of the left anterior wall [5,6], whilst right coronary artery CTO-PCI is more commonly considered for symptom relief, but is unlikely to confer a survival benefit [5]. CTO of the left main coronary artery (LMCA) is an infrequent finding, however in chronic stable non-CTO CAD, left main coronary atery (LMCA) lesions carry the worst prognosis of any coronary lesion, mainly because of the extensive myocardium that is put at risk during intervention [7]. The prognostic significance of concomitant LMCA in CTO-PCI is unknown, however the presence of LMCA disease in CTO patients—in accordance with current guidelines—leads commonly to referral for coronary artery bypass graft (CABG) surgery, although significance of concomitant LM lesions remains unknown [8]. For this purpose, we sought to define clearly the impact of concomitant left main coronary artery diseases on outcome in CTO-PCI in order to guide clinical decision making.

## 2. Methods

### 2.1. Study Population

All consecutive adult patients undergoing PCI for at least one coronary CTO lesion at our tertiary care center between 2005 and 2013 were included in this prospective observational study. All procedures were performed by skilled CTO operators. Indications for CTO-PCI were: (i) inducible myocardial ischemia evaluated by stress echocardiography or myocardial perfusion scintigraphy; (ii) presence of viable myocardium on cardiac magnetic resonance imaging; and (iii) presence of angina symptoms. Patients with acute coronary syndromes, acute infections, and procedural complications (i.e., coronary artery perforation, aortic dissection, tamponade, bleeding, stroke, and surgery related to PCI) were excluded. The study was approved by the local Ethics Committee.

### 2.2. Procedural Characteristics

CTOs were defined as angiographic presence of a total occlusion with Thrombolysis In Myocardial Infarction (TIMI) grade 0 flow and estimated duration of at least 3 months [9]. Retrograde CTO-PCI was defined, if a guidewire was introduced into a collateral channel that supplied the target CTO vessel distal to the lesion. Left main coronary artery disease was defined as CAD of the left main coronary artery. Most importantly in all cases, LM CAD was hemodynamically irrelevant as those patients had either aorto-coronary bypass (ACBP) or preprocedural stenting or non-significant stenosis (≤50%). Procedural success was defined as achievement of <30% residual diameter stenosis within the treated segment and restoration of TIMI grade 3 antegrade flow without the occurrence of complications. Unfractionated heparin was given intravenously at the start of the procedure to maintain an activated clotting time of 250–300 s in patients undergoing antegrade and 300–350 s in patients undergoing retrograde approach. All procedural decisions, including material selection and revascularization strategy, were made at the operator’s discretion.

### 2.3. Clinical Measures and Follow-Up

At study enrollment, medical history, current medication, and electrocardiogram recording were collected. Routine laboratory parameters were analyzed from venous blood samples according to the local laboratory’s routine. All patient data were derived from a standardized follow-up protocol, hospital admission records, the referring physician, or from the outpatient clinic and were entered into a dedicated clinical database followed regularly by outpatient visits or telephone contacts as previously described [10]. Follow-up data were prospectively obtained from hospital readmission, outpatient records, and telephone interview with the patient and/or referring physician. All-cause mortality in 5-years follow-up was primary endpoint. As the secondary study endpoint, a composite endpoint comprising death, non-fatal myocardial infarction (MI), and target lesion vessel revascularization (TVR) was used.

### 2.4. Statistical Methods

Discrete data were presented as count and percentage and analyzed by a χ^2^ test. Continuous data were presented as median and interquartile range (IQR) and compared by the Kruskal–Wallis test. Cox proportional hazard regression analysis was applied. To account for potential confounding effects, we formed a confounder cluster encompassing age, sex, body mass index (BMI), smoking, hypertension, diabetes, New York heart association (NYHA) class, Canadian cardiovascular society (CCS) class, history of myocardial infarction, history of CABG surgery, history of percutaneous coronary intervention, left ventricular (LV) function, multi-vessel disease, successful revascularization, interventional approach (antegrade/retrograde), creatinine, low density lipoprotein (LDL) cholesterol, high density lipoprotein (HDL) cholesterol, hemoglobin, and thrombocyte count.

A stepwise bootstrap resampling procedure including all aforementioned variables was used to identify best-fitting variables for the final multivariable Cox regression model. Five hundred repeats with a *p*-value of 0.05 for selection were performed and variables selected in 80% of all repeats were included in the final cofounder model (i.e., age, hemoglobin and successful revascularization). Additionally, we adjusted for a clinical confounder model encompassing age, LV function, successful revascularization, antegrade/retrograde approach, multi-vessel disease, history of CABG, creatinine, and more than one CTO vessel. The proportional hazards assumption was tested and satisfied in all cases using Schoenfeld residuals. Kaplan–Meier analysis (log-rank test) was applied to assess the time-dependent discriminative power of left main coronary artery disease. Two-sided *p* values <0.05 were used to indicate statistical significance. The STATA11 software package (StataCorp) and SPSS 25.0 (IBM Corp) were used for all analyses.

## 3. Results

### 3.1. Baseline Characteristics

In this contemporary cohort of CTO patients, we enrolled a total of 3860 consecutive patients with a median age of 66 years (IQR57-74). Of those, eighty-three percent (*n* = 3218) were male, with a total of 2282 (59%) presenting in NYHA functional class II and III and a total of 1990 (52%) patients in CCS class II and III. The majority of all patients (98%) were treated via a transfemoral approach. Concomitant right coronary artery (RCA) disease was present in 1800 patients (47%), a history of CABG in 594 patients (15%), and a history of previous PCI in 645 patients (17%). A retrograde CTO wire crossing was performed in 987 (26%) of patients. Within the observational period, 359 patients (10%) died, 580 patients (15%) needed a target vessel revascularization, and 922 patients (34%) experienced a major adverse cardiac event.

Detailed baseline characteristics of the entire study population are displayed in Table 1. Baseline characteristics of total study population (*n* = 3860) according to the presence of significant left main coronary artery disease. Continuous variables are given as medians and interquartile ranges (IQR). Counts are given as numbers and percentages, *p*-values are calculated using Mann–Whitney statistics.

### 3.2. Clinical Characteristics According to Left Main Coronary Artery Disease

Detailed baseline characteristics according to the presence of left main disease are presented in Table 1. In brief, patients with LMCA disease were older (*p* < 0.001), exhibited more often cardiovascular risk factors arterial hypertension (*p* < 0.001), smoking (*p* < 0.001), and diabetes (*p* = 0.036). Furthermore, patients with LMCA disease showed a more complex coronary anatomy as depicted by increased numbers of prior CABG (*p* < 0.001), prior PCI (*p* = 0.017), as well as by more extensive coronary calcification (*p* < 0.001). Overall, we observed a low rate of severe complications with 0.3% of the patients presenting with disabling stroke, 1% with cardiac tamponade, 0.8 % with coronary artery perforation, 2.4% with bleeding requiring transfusion, and 0.8% requiring surgery for a CTO-associated complication.

### 3.3. Left Main Coronary Artery Disease and Outcome

**Figure 1:**
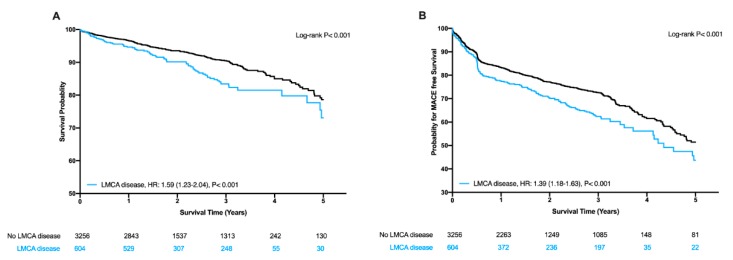
Kaplan–Meier estimates of mortality (**A**) and composite of death, non-fatal myocardial infarction (MI), and target lesion vessel revascularization (TVR) (**B**) according to LMCA disease.

In total, 604 patients (16%) of all patients had left main disease. Of those, 26.8% have had an ACBP for LMCA, 12.5% PCI for LMCA disease, and 60.7% have had non-significant LMCA disease, respectively. Kaplan–Meier analysis demonstrated a significant increase in mortality (Figure 1A, log-rank *p* < 0.001) and the composite secondary endpoint (Figure 1B, log-rank *p* < 0.001) comparing patients with and without left main coronary artery disease. Kaplan–Meier analysis demonstrated a significant increase in mortality (Figure 1A, log-rank *p* < 0.001) and the composite secondary endpoint (Figure 1B, log-rank *p* < 0.001) comparing patients with and without left main coronary artery disease. In the Cox regression analysis, we observed a crude HR 1.59 (95%CI 1.23–2.04, *p* < 0.001) for patients with left main disease as compared to patients without. The results remained unchanged after adjustment for the bootstrap-selected confounder model with an adjusted HR of 1.32 (95%CI 1.03–1.70, *p* = 0.031). In line with this, we found after adjustment for the clinical confounder model an adjusted HR of 1.32 (95%CI 1.006–1.73, *p* = 0.045) for patients with left main disease (Table 2). Similarly, we found an association for the secondary endpoint, a composite of death, non on-fatal myocardial infarction (MI), and target lesion vessel revascularization (TVR) with a crude HR of 1.39 (95%CI 1.18–1.63, *p* < 0.001) for patients with left main disease as compared to patients without. Detailed results of the multivariable analysis are displayed in Table 2.

### 3.4. Left Main Coronary Artery Disease and Outcome According to Target Vessel

To elucidate the influence coronary anatomy, we divided our study cohort according to the CTO target vessel and repeated the crude and multivariable Cox regression model assessing the influence left main coronary artery disease. We found that neither in the univariable nor in the adjusted Cox regression models left main coronary artery disease was associated with mortality or the secondary endpoint in patients undergoing CTO-PCI for LAD disease (Table 3 and Table 4).

In patients with RCA CTO, we observed a crude HR of 1.59 (95%CI 1.08–2.35, *p* = 0.018) for mortality and 1.36 (95%CI 1.06–1.74, *p* = 0.014) for our secondary endpoint. However, in the clinical and the bootstrap-adjusted model, this association failed to show significance for both the primary and the secondary endpoint (Table 3 and Table 4). In patients with CX (circumflex artery) CTO, we observed a crude HR of 1.99 (95% CI 1.26–3.14, *p* = 0.005) for mortality and 1.72 (95%CI 1.28–2.32, *p* < 0.001) for the secondary endpoint, which remained virtually unchanged in the clinical and the bootstrap-adjusted confounder model (Table 3 and Table 4).

## 4. Discussion

This large prospective, observational, single center registry of 3860 consecutive CTO revascularizations explores, for the first time, the influence on concomitant left main coronary artery disease and outcome after CTO-PCI. We found not only that LMCA is a strong and independent risk factor for unfavorable outcome, but that excess mortality is largely driven by anatomical features i.e., CTO of the circumflex artery.

### 4.1. Risk Profile of Left Main Coronary Artery Disease in CTO Patients

Our study revealed that 16% of all patients (*n* = 604) with CTOs additionally exhibited significant left main coronary artery disease, which is in line with already published incidence rates of 2.5%−17.8% [11,12]. Patients with left main disease were significantly older (*p* < 0.001) and exhibited more often cardiovascular risk factors like smoking (*p* < 0.001), diabetes (*p* = 0.036), and arterial hypertension (*p* < 0.001). In tandem with the higher baseline risk for CAD, those patients were more prone to procedural complexity as depicted by higher rates of prior CABG (*p* < 0.001) or PCI (*p* = 0.017) and more extensive coronary calcification (*p* < 0.001).

### 4.2. Long Term Clinical Outcomes in Patients with Left Main Coronary Artery Disease

The left main coronary artery supplies 75% of the LV in right dominant circulation and 100% in left dominant circulation [13]. Not surprisingly, we found that left main coronary artery disease is a significant and independent predictor of increased mortality and major adverse cardiac events (MACE) following CTO-PCI. One might argue that the observed difference is the sequela of the complexity of coronary arthrosclerosis in patients with LM disease, as depicted by the increased rates of prior CABG, PCI, and the more extensive coronary calcification. From this point of view, the observed association of unfavorable outcome and left main coronary artery disease only reflects the extent of atherosclerosis and lesion complexity and defines the left main coronary artery to be a simple branch of the coronary tree among others.

However, the exclusive role in blood supply for the left ventricle and the characteristic histology of the LMCA (lack of adventitia and the substantial smooth muscle and elastic tissue) makes the LMCA unique among all coronary arteries [14]. Therefore, to truly define the predictive role of LMCA disease, we included not only well-established clinical risk factors for cardiovascular mortality, but also variables that depict the atherosclerotic burden and lesions complexity of those patients into our Cox proportional hazard models to adjust the risk. In line with our unadjusted model, we observed that the left main coronary artery disease is a strong and independent risk factor for mortality (Table 2). This finding further underpins the substantial role of the LMCA and further defines LMCA stenosis as an independent risk factor and not as a sign of more advanced disease. Furthermore, we observed that LMCA disease anatomy (ACBP, PCI, or non-significant stenosis) had no further additive effect on mortality. Concomitantly with mortality, we observed analogous results for our secondary study endpoint (Table 2). However, the present findings should not be interpreted with regard to encourage CABG surgery as an alternative revascularization strategy in all CTO patients with concomitant LMCA disease, as this strategy also harbors a significant increased risk in CTO patients [15]. Strength of the present analysis is that we are the first to clearly define CTO coronary anatomies in which LMCA disease leads to excess mortality and those in which not. Especially in patients with CTO of the LAD or the RCA, concomitant left main coronary artery disease does not exacerbate periprocedural risk for mortality and MACE, suggesting PCI revascularization as strategy of choice.

Overall, the exponential developments in CTO percutaneous coronary intervention equipment, crossing techniques, and operator experience, in conjunction with advanced periprocedural adjunctive pharmacotherapies, makes CTO PCI also in high risk patients still a valuable treatment option [16].

### 4.3. Outcomes in Patients with Left Main Coronary Artery Disease According to Target Vessel

To further define a high risk patient population that among those patients with LMCA disease, we divided our study cohort according to the CTO target vessel and repeated the crude and multivariable Cox regression model assessing the influence of left main coronary artery disease.

We found for both mortality and our secondary endpoint that those patients with a chronically occluded circumflex artery show the highest risk of unfavorable outcome (Table 3 and Table 4). This finding goes in line with previous observations that CTO procedures of the circumflex artery is associated with a lower rate of procedural success and higher rates of MACE [17]. This higher risk results from several possible mechanisms. For instance, the circumflex artery is more tortious, hindering advancing attempts and decreasing the torque response as well as the steerability to the occlusion site [18]. Additionally, LCX CTOs show lower numbers of collaterals, limiting use of the retrograde approach [17]. Finally, post interventional ischemic complications are more likely to be underestimated, as the LCX-supplied territory is mostly an electrocardiographically silent area [17,18].

### 4.4. Limitations

The present study was an observational study; which always harbors the risk that the observed differences (which were adjusted for in multivariate Cox proportional analysis) are accompanied by unmeasured differences (e.g., adherence to evidence-based medical therapies during follow-up) which might confound the associations between the measured variables and the clinical outcome. Presence of LMCA disease was based solely on angiographic grading and not further validated by intravascular imaging. In the majority of all patients, a femoral and not radial approach was chosen, which might has influenced mortality in the present analysis. The primary outcome parameter mortality was robust and obtained prospectively from hospital readmission, outpatient records, and telephone interview with the patient and/or referring physician. As in the present study, SYNTAX score was not available in all patients, the multivariable regression analysis could not be corrected for this anatomic risk score.

## 5. Conclusions

In summary, in this large contemporary cohort of CTO patients, we show that left main coronary artery disease is associated with an unfavorable long-term outcome. While anatomical features such as CTO of the circumflex artery anticipate high risk procedures, LAD or RCA CTOs in conjunction with left main coronary artery disease are not associated with unfavorable long-term prognosis, rendering CTO-PCI as a valuable therapeutic option in this setting.

## Figures and Tables

**Table 1:** Baseline characteristics of total study population (*n* = 3860) according to the presence of significant left main coronary artery disease. Continuous variables are given as medians and interquartile ranges (IQR). Counts are given as numbers and percentages, *p*-values are calculated using Mann–Whitney statistics.

	Total Study Population (*n* = 3860)	LMCA Disease (*n* = 604)	No LMCA (*n* = 3256)	*p*-Value
Baseline Characteristics				
Age, median years (IQR)	66 (57–74)	68 (60–75)	65 (57–73)	<0.001
Male sex, *n* (%)	3218 (83)	517 (86)	2701 (83)	0.116
BMI, kg/m^2^(IQR)	27.8 (25.2–30.7)	27.8 (25.4–30.9)	27.7 (25.2–30.6)	0.742
Smoking, *n* (%)	731 (19)	83 (14)	648 (20)	<0.001
Hypertension, *n* (%)	3202 (83)	533 (88)	2669 (82)	0.001
Diabetes, *n* (%)	1111 (29)	199 (33)	912 (28)	0.036
Hypercholesterolemia, *n* (%)	3270 (85)	522 (86)	2748 (84)	0.953
Family history of CAD, *n* (%)	1410 (37)	231(38)	1179 (36)	0.839
Previous myocardial infarction, *n* (%)	1161 (30)	194 (32)	967 (30)	0.466
Previous CABG, *n* (%)	594 (15)	162 (27)	432 (13)	<0.001
Previous PCI, *n* (%)	645 (17)	121 (20)	524 (16)	0.017
NYHA functional class				0.235
NYHA II	1444 (37)	218 (36)	1226 (38)	
NYHA III	838 (22)	147 (24)	691 (21)	
NYHA IV	123 (3)	19 (3)	104 (3)	
CCS class				0.473
CCS II	1143 (30)	186 (31)	957 (30)	
CCS III	847 (22)	127 (21)	720 (22)	
CCS IV	615 (16)	108 (18)	507 (16)	
Reduced LV function (LVEF < 40%), *n* (%)	242 (6)	48 (8)	194 (6)	0.102
J-CTO score (IQR)	2 (1–3)	2 (1–3)	2 (1–3)	<0.001
Creatinine, mg/dL (IQR)	1.0 (0.9–1.2)	1.0 (0.9–1.2)	1.0 (0.9–1.1)	0.114
LDL cholesterol, mg/dL (IQR)	108 (83–139)	106 (82–139)	108 (84–139)	0.682
HDL cholesterol, mg/dL (IQR)	47 (39–56)	47 (39–54)	47 (39–57)	0.746
Hemoglobin (g/dL), median (IQR)	14.4 (13.4–15.3)	14.3 (13. 1–15.2)	14.4 (13.4–15.3)	0.019
Retrograde approach, *n* (%)	987 (26)	168 (28)	819 (25)	0.178
Extensive coronary calcification, *n* (%)	1087 (28)	202 (33)	885 (27)	<0.001
Amount of contrast dye used (mL), median (IQR)	300 (200–400)	300 (220–420)	300 (200–400)	0.008
Nominal stent diameter (mm), median (IQR)	3.00 (2.75–3.50)	3.00 (2.75–3.50)	3.00 (2.75–3.50)	0.267
Concomitant RCA disease, *n* (%)	1800 (46.6)	271 (44.9)	1529 (47)	0.344
Procedural success *n* (%)	3257 (84)	494 (82)	2763 (85)	0.056

LMCA, left main coronary artery; BMI, body mass index; CAD, coronary artery disease; CABG, coronary artery bypass graft; PCI, percutaneous coronary intervention; NYHA, New York heart association; CCS, Canadian cardiovascular society; LVEF, left ventricular ejection fraction J-CTO, Japan CTO; IQR, Interquartile range LDL, low density lipoprotein; HDL, high density lipoprotein; RCA, right coronary artery.

**Table 2:** Crude and multivariable Cox regression model assessing the impact of left main disease on outcome. The secondary endpoint represents a composite of death, non-fatal myocardial infarction (MI), and target lesion vessel revascularization (TVR).

	Univariable Model		Multivariable Model *		Bootstrap-Adjusted Confounder Model ^†^	
All-Cause Mortality	Crude HR (95% CI)	*p*-Value	Adj. HR (95% CI)	*p*-Value	Adj. HR (95% CI)	*p*-Value
LMCA disease	1.59 (1.23–2.04)	<0.001	1.32 (1.006–1.73)	0.045	1.32 (1.03–1.70)	0.031
Secondary Endpoint						
LMCA disease	1.39 (1.18–1.63)	<0.001	1.25 (1.06–1.48)	0.009	1.33 (1.14–1.57)	<0.001

* age, successful revascularization, interventional approach, three vessel disease, history of CABG, creatinine, and reduced left ventricular function. † adjusted for: age, hemoglobin, and successful revascularization. Adj. HR, adjusted hazard ratio; CI, confidence interval.

**Table 3:** Crude and multivariable Cox regression model assessing the impact of LMCA disease and mortality divided target vessels.

	Univariable Model		Multivariable Model *		Bootstrap-Adjusted Confounder Model ^†^	
	Crude HR (95% CI)	*p*-Value	Adj. HR (95% CI)	*p*-Value	Adj. HR (95% CI)	*p*-Value
LAD-CTO	1.41 (0.86–2.31)	0.175	1.30 (0.76–2.24)	0.338	1.15 (0.70–1.90)	0.581
RCA-CTO	1.59 (1.08- 2.35)	0.018	1.32 (0.87–2.01)	0.197	1.17 (0.79–1.73)	0.448
CX-CTO	1.99 (1.26–3.14)	0.005	1.65 (1.02–2.68)	0.042	2.01 (1.27–3.16)	0.003

* age, successful revascularization, interventional approach, three vessel disease, history of CABG, creatinine, and reduced left ventricular function. † adjusted for: age, hemoglobin, and successful revascularization. LAD-CTO, left anterior descending chronic total occlusion, CX-circumflex artery chronic total occlusion

**Table 4:** Crude and multivariable Cox regression model assessing the impact of LMCA disease and composite of death, non on-fatal myocardial infarction (MI), and target lesion vessel revascularization (TVR) divided target vessels.

	Univariable Model		Multivariable Model *		Bootstrap-Adjusted Confounder Model ^†^	
	Crude HR (95%CI)	*p*-Value	Adj. HR (95%CI)	*p*-Value	Adj. HR (95%CI)	*p*-Value
LAD-CTO	1.24 (0.90–1.72)	0.189	1.12 (0.80–1.58)	0.509	1.26 (0.98–1.61)	0.069
RCA-CTO	1.36 (1.06–1.74)	0.014	1.16 (0.89–1.50)	0.263	1.17 (0.84–1.62)	0.351
CX-CTO	1.72 (1.28–2.32)	<0.001	1.75 (1.28–2.34)	<0.001	1.78 (1.32–2.39)	<0.001

* age, successful revascularization, interventional approach, three vessel disease, history of CABG, creatinine, and reduced left ventricular function. **†** adjusted for: age, hemoglobin, and successful revascularization.
